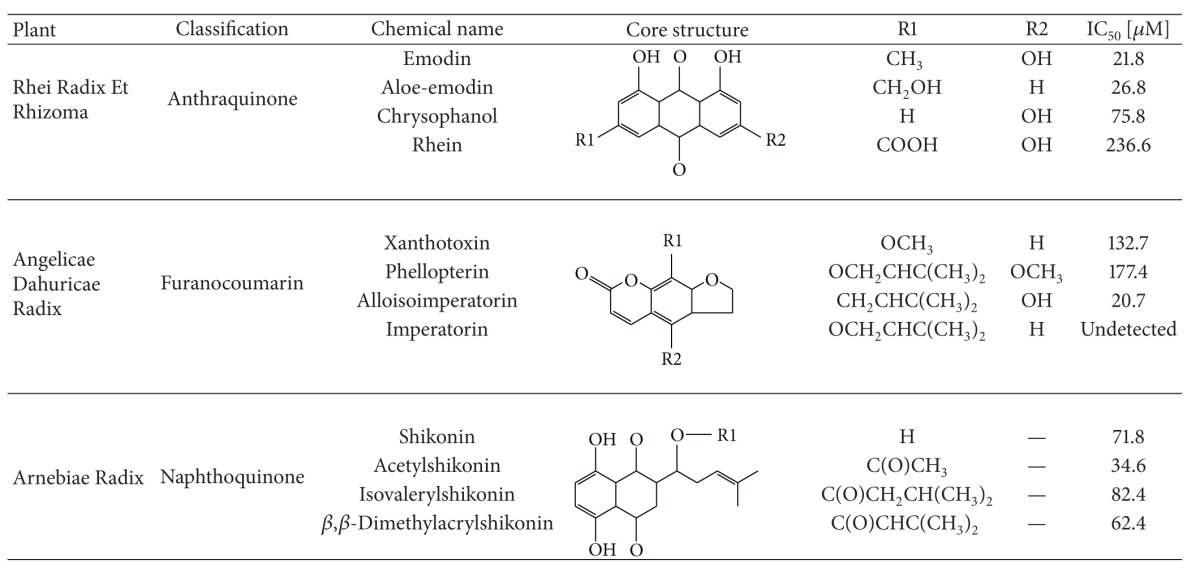# Acetylshikonin, a Novel AChE Inhibitor, Inhibits Apoptosis via Upregulation of Heme Oxygenase-1 Expression in SH-SY5Y Cells

**DOI:** 10.1155/2013/937370

**Published:** 2013-11-05

**Authors:** Yan Wang, Wen-Liang Pan, Wei-Cheng Liang, Wai-Kit Law, Denis Tsz-Ming Ip, Tzi-Bun Ng, Mary Miu-Yee Waye, David Chi-Cheong Wan

**Affiliations:** School of Biomedical Sciences, The Chinese University of Hong Kong, Shatin, New Territories, Hong Kong

## Abstract

Acetylcholinesterase inhibitors are prominent alternative in current clinical treatment for AD patients. Therefore, there is a continued need to search for novel AChEIs with good clinical efficacy and less side effects. By using our in-house natural product database and AutoDock Vina as a tool in docking study, we have identified twelve phytochemicals (emodin, aloe-emodin, chrysophanol, and rhein in Rhei Radix Et Rhizoma; xanthotoxin, phellopterin, alloisoimperatorin, and imperatorin in Angelicae dahuricae Radix; shikonin, acetylshikonin, isovalerylshikonin, and *β*,*β*-dimethylacrylshikonin in Arnebiae Radix) as candidates of AChEIs that were not previously reported in the literature. In addition to AChEI activity, a series of cell-based experiments were conducted for the investigation of their neuroprotective activities. We found that acetylshikonin and its derivatives prevented apoptotic cell death induced by hydrogen peroxide in human and rat neuronal SH-SY5Y and PC12 cells at 10 *μ*M. We showed that acetylshikonin exhibited the most potent antiapoptosis activity through the inhibition of the generation of reactive oxygen species as well as protection of the loss of mitochondria membrane potential. Furthermore, we identified for the first time that the upregulation of heme oxygenase 1 by acetylshikonin is a key step mediating its antiapoptotic activity from oxidative stress in SH-SY5Y cells.

## 1. Introduction

Alzheimer's disease (AD) is one of the most devastating neurodegeneration diseases characterized by progressive memory loss and cognitive dysfunction in the aging population. Although beta-amyloid aggregation and fibrillar tau-tangles have been identified as the major pathogenesis markers in AD patients and they are now promising targets for drug development, there is still no available drug against these targets (reviewed in [1–3]). Therefore, acetylcholinesterase inhibitors (AChEIs) are alternative option in current clinical treatment, and there is a continued need to search for novel AChEIs with less side effect to treat AD [4].

Synthetic compounds are now a central focus when searching any AChEIs. Many of these AChEIs potently inhibited the enzyme at the nanomolar level [5–7]. However, not much information regarding the potency and efficacy of these AChEIs in animal study or clinical trials can be gathered, due to the fact that the potency of AChEIs inhibition may not correlate with their neuroprotection efficacy due to their increases in cellular toxicity. It is supported by the recent study that the role of AChEIs against AD might be far beyond its AChE inhibition that enhances neuronal transmission acetylcholine [8]. Abundant evidence from *in vitro* and *in vivo* studies has demonstrated that AChEIs exhibited remarkably neuroprotective effects through attenuation of oxidative stress and enhancement of antioxidant status [9, 10]. Therefore, both anti-AChE activity and antioxidative stress should be considered when searching novel AChEIs as drugs to treat AD. 

In recent years, *in silico* virtual drug screening became a preferred approach to screen novel compounds given that the structures of molecular targets (enzymes or receptors) are determined. The high-throughput docking screen can provide possible candidates for further biomedical validation so as to reduce the time and cost of research and development in drug discovery (reviewed in [11]). The availability of the structure of AChEs has provided the opportunity of widespread *in silico* screening of novel AChEIs [12–18]. The objective of the present study is to compile a comprehensive database from natural herbs in which the key constituents have been chemically characterized. It was inspired by the fact that the natural AChEI, galantamine, the FDA approved drug to treat mild-to-moderate AD, is a natural alkaloid that has only mild AChEI activity but strong neuroprotective efficacy [19]. Using this database, we have successfully identified some groups of phytochemicals that have mild AChEI activity but showed very promising neuroprotection in neuronal cell cultures induced by oxidative damages.

## 2. Materials and Methods

### 2.1. Molecular Docking Screening

For ligands library establishment, approximately 8,000 phytochemicals were compiled based on selected reference books. The SMILE format of phytochemicals was compiled from Pubchem (http://pubchem.ncbi.nlm.nih.gov/) or Scifinder (https://www.cas.org/products/scifinder/). The SMILES format of compounds was converted to PDB format by CORINA online service (http://www.molecular-networks.com/online_demos/corina_demo/). The PDB format of compounds was then converted to PDBQT format by AutoDock Tools 1.5.6 (The Scripps Research Institute, CA, USA). For receptor preparation, the crystal structure of human AChE was obtained from the Protein Data Bank (PDB 1B41). Both ligands and water molecules in 1B41 were removed by Chimera 1.7mac (UCSF Resource for Biocomputing, Visualization, and Informatics, CA, USA). The modified 1B41 was converted to PDBQT format by AutoDock Tools 1.5.6 (The Scripps Research Institute, CA, USA) for docking screening. The docking parameters were set as previous study with default values, and the size of grid box was set as 20 Å × 20 Å × 20 Å for encompassing catalytic site. The molecular docking screening was performed by AutoDock Vina v.1.0.2 (The Scripps Research Institute, CA, USA).

### 2.2. Reagents and Antibodies

2′,7′-Dichlorodihydrofluorescein diacetate (H_2_DCF-DA), pentahydrate (bis-benzimide) (Hoechst 33258), and 3,6-diamino-9-(2-(methoxycarbonyl) phenyl, chloride (Rhodamine 123) were obtained from Invitrogen (Carlsbad, CA, USA). Acetylthiocholine iodide (ATCI), 5′,5-dithio-bis-(2-nitrobenzoate) (DTNB), zinc protoporphyrin IX (ZnPP), H_2_O_2_, and all other chemicals used in this study were purchased from Sigma (St. Louis, MO, USA). All cell culture reagents were obtained from Invitrogen (Carlsbad, CA, USA). Antibodies against p53, Bax, Bcl-2, caspase-3, and beta-actin were purchased from Cell Signaling Technology (Danvers, MA, USA). Antibody against HO-1 was obtained from Santa Cruz Biotechnology (Santa Cruz, CA, USA). Shikonin, acetylshikonin, beta, beta-dimethylacrylshikonin, isovalerylshikonin, xanthotoxin, phellopterin, imperatorin, and alloisoimperatorin were obtained from Apin Chemicals Ltd (Oxfordshire, UK). Emodin, aloe-emodin, rhein. and chrysophanol were obtained from National Institutes for Food and Drug Control (Beijing, China). The test chemicals were dissolved in distilled water and dimethyl sulfoxide (DMSO); the final concentration of DMSO was less than 0.1%.

### 2.3. AChE Assay

Candidate phytochemicals dissolved in DMSO were tested for AChE inhibitory activity by the Ellman assay with minor modifications [20]. Ten *μ*L of human recombinant AChE (prepared in-house [20]) and 1 *μ*L of drug were added into 190 *μ*L of PBS buffer (100 mM, pH 7.4) and incubated in a 96-well plate at 37°C for 10 min. Then 25 *μ*L of 12.5 mM ATCI and 25 *μ*L of 10 mM DTNB were premixed and added into each well. After 10 min incubation with the substrate, the optical densities were measured in a 96-well plate reader at 412 nm. The optical density was inversely proportional to the inhibitory activity. By contrast, a blank control without the tested compound was also performed in parallel; the normal hydrolytic rate of the enzyme can be represented by the blank control. Each assay was performed in triplicate.

The percentage inhibitory activities of the various compounds were calculated by comparison with the positive control and the blank control. The formula was shown as follows: percent of inhibitory activity of the compound = (1 − absorbance of sample/absorbance of blank control)/(1 − absorbance of positive control/absorbance of blank control) × 100%. Data analysis was performed with Prism software. Inhibitory effects were expressed as IC_50_ value calculated by regression analysis.

### 2.4. Cell Cultures

Human neuroblastoma SH-SY5Y cells were from ATCC (Manassas, VA, usa) and maintained in DMEM/F-12 containing 10% FBS and maintained at 37°C with 95% humidified air and 5% CO_2_. Rat adrenal medulla pheochromocytoma PC12 cells were from ATCC (Manassas, VA, USA) and maintained in DMEM containing 10% FBS at 37°C with 95% humidified air and 5% CO_2_. 

### 2.5. Cell Viability Assay

MTT colorimetric assay was performed to determine the cell viability. Cells were seeded in 96-well plates at a density of 5 × 10^3^ cells/well and treated with test chemicals at desired concentration at 37°C for 12 hours. Subsequently, cells were stimulated with H_2_O_2_ (500 *μ*M) for 4 hours. After the exposure period, the cells were incubated with 20 *μ*L MTT (5 mg/mL) for 4 h. The cells were eluted with DMSO and quantified with a spectrophotometer (Ultramark Microplate Reader, Bio-Rad) at a wavelength of 590 nm.

### 2.6. Nuclear Staining with Hoechst 33258

SH-SY5Y cells and PC12 cells (1 × 10^4^ cells/well) in 24-well plates were preincubated with or without test chemicals for 12 hours and subsequently stimulated with H_2_O_2_ for the 4 hours. The nuclear morphology of apoptotic cells was measured by Hoechst 33258 nuclear staining according to the manufacturer's instructions. The nuclear morphological change was observed under a fluorescence microscope (Nikon Live Cell Imaging System Ti-E, Japan) using excitation/emission of 360/460 nm.

### 2.7. Intracellular Reactive Oxygen Species (ROS) Measurement

The cells were treated with desired concentration of acetylshikonin for 12 hours, and then the cells were stained with H_2_DCF-DA (10 *μ*M) for 30 min. After 30 min staining, cells were stimulated with H_2_O_2_ (500 *μ*M) for 2 h, and the fluorescence intensity of H_2_DCF-DA was measured/detected by a fluorescence spectrophotometer (M1000, TECAN, Austria GmbH, Austria) using excitation/emission of 485/530 nm and a fluorescence microscope (Nikon Live Cell Imaging System Ti-E, Japan) using excitation/emission of 490/530 nm, respectively. Fluorescence intensity of each group was normalized to the control group.

### 2.8. Measurement of Mitochondrial Membrane Potential

The cells were treated with desired concentration of acetylshikonin for 12 hours, and then the cells were stimulated with H_2_O_2_ (500 *μ*M) for 2 h. Rhodamine 123 (2 *μ*M) was added to cells after the treatment for 30 min at 37°C. Fluorescence intensity of Rhodamine 123 was measured/detected by a fluorescence spectrophotometer (M1000, TECAN, Austria GmbH, Austria) using excitation/emission of 485/530 nm and a fluorescence microscope (Nikon Live Cell Imaging System Ti-E, Japan) and using excitation/emission of 490/530 nm, respectively. Fluorescence intensity of each group was normalized to the control group.

### 2.9. Western Blot Assay

Proteins in the total cell lysate were separated by 10% SDS polyacrylamide gel electrophoresis and electrotransferred to a polyvinylidene difluoride membrane (Immobilon-P membrane; Millipore, Bedford, MA, USA). After the blot was blocked in a solution of 5% bovine serum albumin, membrane was incubated overnight with primary antibodies against Bcl-2, Bax, Caspase-3, p53, HO-1, or beta-actin followed by incubation with horseradish peroxidase-conjugated secondary antibodies for 1 h. Specific bands were detected with ECL-plus western blotting detection reagent (GE Healthcare Bio-Sciences) and photographed with FujiFilm LAS-3000 (Fujifilm, Tokyo, Japan). 

### 2.10. Statistics

Statistical significance was determined using the One-Way ANOVA (GraphPad Software, CA, USA). The results are presented as the means ± SEM. The significance was accepted when *P* value was <0.05.

## 3. Results

### 3.1. Potential AChE Inhibitors from Natural Products Were Identified by Molecular Docking Screen

Using the natural product database and AutoDock vina for screening, we have identified 12 phytochemicals reportedly (emodin, aloe-emodin, chrysophanol, and rhein from anthraquinone fraction in RHEI RADIX ET RHIZOMA; xanthotoxin, phellopterin, alloisoimperatorin, and imperatorin from furanocoumarin fraction in ANGELICAE DAHURICAE RADIX; shikonin, acetylshikonin, isovalerylshikonin, and *β*,*β*-dimethylacrylshikonin from naphthoquinone fraction in ARNEBIAE RADIX) which can act as AChEIs. Huperzine A, the positive control, exhibited the highest docking score in the ranking list. It is noted that Trp86 is the key residue interacting with all AChEIs through *π*-*π* interaction in docking simulation, which is consistent with the key role of Trp86 in the catalytic pocket of AChE (Table 1) [21]. *In vitro* validation demonstrated that anthraquinones from RHEI RADIX ET RHIZOMA were the strongest AChEIs (Table 2). The inhibition of emodin, aloe-emodin, chrysophanol, and rhein on human AChE showed different degrees of concentration-dependent inhibition. Among these, emodin and aloe-emodin were more potent with IC_50_ 21.80 *μ*M and 26.76 *μ*M, respectively. The other anthraquinones exhibited relatively weak inhibitory effects on AChE activity. Alloisoimperatorin is most potent anti-AChE chemicals with IC_50_ 20.7 *μ*M in furanocoumarin fraction in ANGELICAE DAHURICAE RADIX. In addition, acetylshikonin is the most potent anti-AChE chemicals with IC_50_ 34.6 *μ*M in naphthoquinone fraction in ARNEBIAE RADIX.

### 3.2. The Effects of H_2_O_2_ or AChE Inhibitors from Natural Products on Cell Viability in SH-SY5Y or PC12 Cells

**Figure 1:**
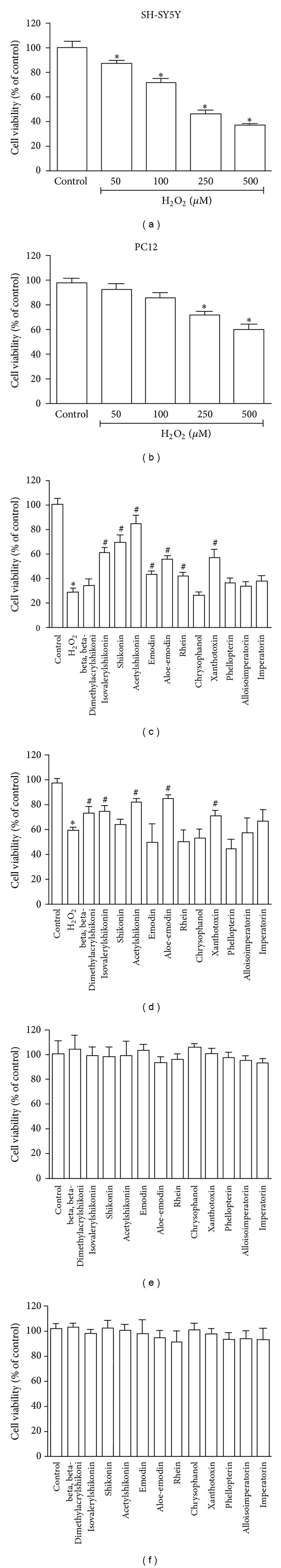
Potential AChE inhibitors of natural products attenuated H_2_O_2_-induced cell death in both SH-SY5Y and PC12 cells. SH-SY5Y (a) or PC12 (b) cells were cultured with desired concentration of H_2_O_2_ for 4 hours; the cell viability was detected by MTT. Data shown are means ± SEM of results from independent experiments in triplicate. *P < 0.05 compared with control cells. SH-SY5Y (c) or PC12 (d) cells were incubated with different potential AChE inhibitors (10 *μ*M) for 12 hours and then stimulated with H_2_O_2_ (500 *μ*M) for 4 hours, and the viability was detected by MTT. Data shown are means ± SEM of results from independent experiments in triplicate. *P < 0.05 compared with control cells; ^#^ *P* < 0.05 compared with H_2_O_2_-stimulated cells. For cytotoxicity test, SH-SY5Y (e) or PC12 (f) cells were incubated with different potential AChE inhibitors (10 *μ*M) for 12 hours, and the viability was detected by MTT. Data shown are means ± SEM of results from independent experiments in triplicate. *P < 0.05 compared with control cells.

H_2_O_2_-induced cytotoxicity in both SH-SY5Y and PC12 cells was treated with various concentrations of H_2_O_2_ (50–500 *μ*M) for 4 hours, and the subsequent cell viability was measured by MTT assay. As shown in Figures 1(a) and 1(b), H_2_O_2_ at concentration of 500 *μ*M led to approximately half-maximal cell death (60% cell death in SH-SY5Y cells and 40% cell death in PC12 cells). Therefore, this concentration was selected to evaluate the potential protective effects of AChE inhibitors from natural products on H_2_O_2_-stimulation oxidative stress and apoptosis in SH-SY5Y and PC12 cells. After pretreatment with test chemicals, the cells were exposed to H_2_O_2_ for 4 hours and applied to MTT assay. Results showed that H_2_O_2_-induced cell death was statistically attenuated by seven test chemicals at 10 *μ*M in SH-SY5Y cells (Figure 1(c)). In PC12 cells, five chemicals rescued H_2_O_2_-induced cell death (Figure 1(d)). Notably, acetylshikonin-treated cells exhibited the highest viability during H_2_O_2_ stimulation, indicating that acetylshikonin might be the strongest neuroprotective candidate among these potential AChE inhibitors. Thus, study in the latter part will be focused on neuroprotective effects of acetylshikonin on H_2_O_2_-induced cell apoptosis in both SH-SY5Y and PC12 cells.

### 3.3. Acetylshikonin Attenuated H_2_O_2_-Induced Cell Death with Dose-Dependent Manner in SH-SY5Y and PC 12 Cells

**Figure 2:**
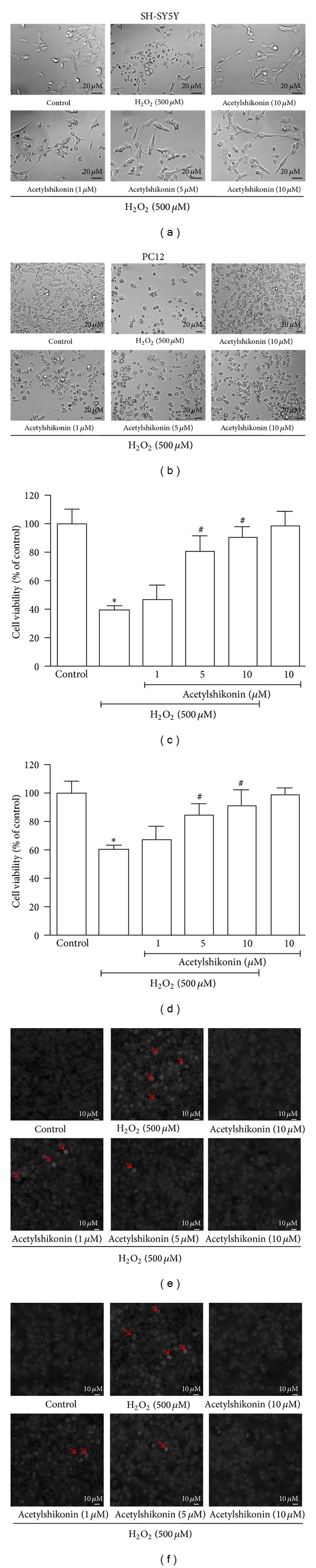
Acetylshikonin attenuated H_2_O_2_-induced cell death and apoptosis in both SH-SY5Y and PC12 cells with dose-dependent manner. SH-SY5Y or PC12 cells were incubated with desired concentration of acetylshikonin for 12 hours and then stimulated with H_2_O_2_ (500 *μ*M) for 4 hours, and the change of cell morphology ((a) SH-SY5Y; (b) PC12), cell viability ((c) SH-SY5Y; (d) PC12), and change of nuclear morphology ((e), SH-SY5Y; (f), PC12) were measured, respectively. Data shown are means ± SEM of results from independent experiments in triplicate. *P < 0.05 compared with control cells; ^#^ *P* < 0.05 compared with H_2_O_2_-stimulated cells.

H_2_O_2_ was a strong peroxide and it significantly induced cell death (*P* < 0.05) and caused morphology change, which were dose dependently attenuated by acetylshikonin (1–10 *μ*M) in both SH-SY5Y (Figures 2(a) and 2(c)) and PC12 (Figures 2(b) and 2(d)) cells. Meanwhile, the cytotoxic potential of acetylshikonin was also tested at 10 *μ*M. Cytotoxicity of acetylshikonin was not observed at used dosage in MTT assay and no morphological change was found. To evaluate the protective effects of acetylshikonin on H_2_O_2_-induced apoptosis, the nuclear morphological observation was measured by Hoechst 33258 staining. As shown in Figures 2(e) and 2(f), H_2_O_2_ stimulation resulted in cell shrinkage and nuclear condensation, which were indicated by red arrows. However, this morphological change was dramatically ameliorated by acetylshikonin in both SH-SY5Y and PC12 cells.

### 3.4. Acetylshikonin Attenuated H_2_O_2_-Induced ROS Generation and Mitochondrial Membrane Potential Loss with Dose-Dependent Manner in SH-SY5Y and PC 12 Cells

**Figure 3:**
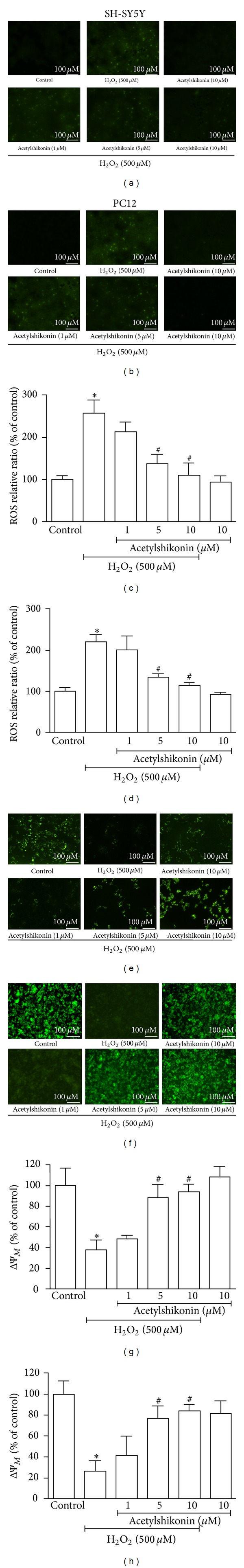
Acetylshikonin attenuated H_2_O_2_-induced ROS generation and mitochondrial membrane potential loss in both SH-SY5Y and PC12 cells with dose-dependent manner. The cells were treated with desired concentration of acetylshikonin for 12 hours and then stimulated with H_2_O_2_ (500 *μ*M) for 2 hours, and the ROS generation and mitochondrial membrane potential loss were detected by H_2_DCF-DA and Rhodamine 123 staining, respectively. Representative photograph of ROS generation in SH-SY5Y (a) or PC12 (b) cells was taken by fluorescence microscope; the quantitative analysis of ROS generation in SH-SY5Y (c) or PC12 (d) cells was measured by fluorescence spectrophotometer. Representative photograph of mitochondrial membrane potential loss in SH-SY5Y (e) or PC12 (f) cells was taken by fluorescence microscope; the quantitative analysis of mitochondrial membrane potential loss in SH-SY5Y (g) or PC12 (h) cells was measured by fluorescence spectrophotometer. Data shown are means ± SEM of results from independent experiments in triplicate. *P < 0.05 compared with control cells; ^#^ *P* < 0.05 compared with H_2_O_2_-stimulated cells.

As shown in Figures 3(a) and 3(b), H_2_O_2_-treated cells exhibited bright green fluorescence while the fluorescence did not appear in the control cells, indicating that total intracellular ROS was significantly increased after H_2_O_2_ stimulation. In contrast, acetylshikonin reduced H_2_O_2_-induced bright green fluorescence at 5 and 10 *μ*M, representing that the ROS generation was also diminished. The quantitative analysis was consistent with microscopic observation; H_2_O_2_-induced ROS generation was statistically declined by pretreatment with acetylshikonin in a dose-dependent manner (Figures 3(c) and 3(d)). 

Mitochondrial depolarization is the critical event in oxidant-induced apoptosis as stated before [22], and the effect of acetylshikonin on H_2_O_2_-induced mitochondrial membrane potential (MMPs) loss was detected by Rhodamine 123 staining. As shown in Figures 3(e) and 3(f) of representative pictures, the control cells exhibited bright green fluorescence, whereas stimulation of H_2_O_2_ cells only showed blank background, reflecting the loss of mitochondrial membrane potentials. Notably, pretreatment with acetylshikonin significantly attenuated the H_2_O_2_-induced MMPs loss. For quantitative analysis, the MMPs were further detected by fluorescence spectrometer. As shown in Figures 3(g) and 3(h), H_2_O_2_ stimulation statistically reduced MMPs, which was rescued by acetylshikonin in a dose-dependent manner in both SH-SY5Y and PC12 cells.

### 3.5. Acetylshikonin Modulated H_2_O_2_-Induced Apoptosis-Related Protein Expression in Both SH-SY5Y and PC12 Cells

**Figure 4:**
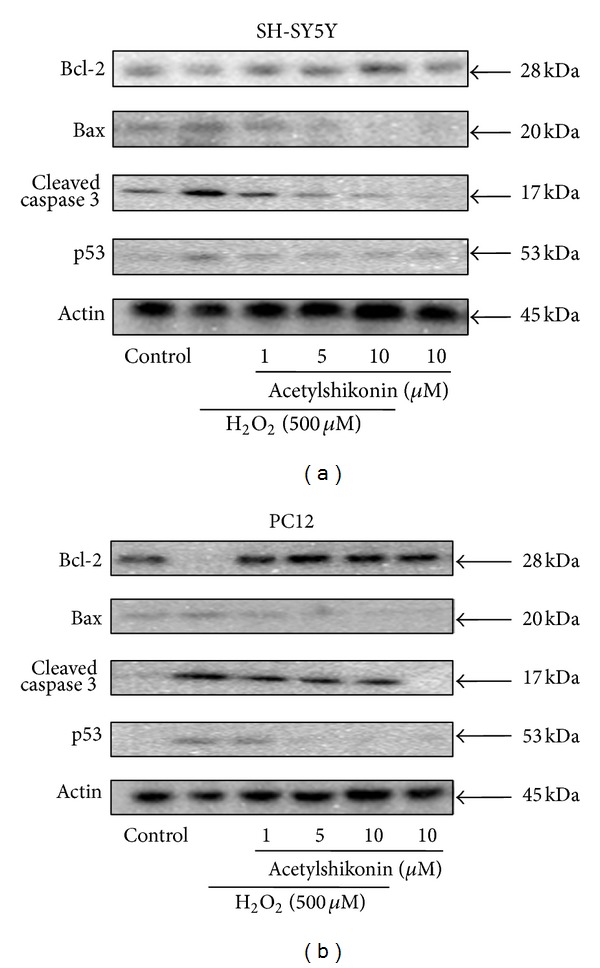
Acetylshikonin modulated H_2_O_2_-induced apoptosis-related protein expression in both SH-SY5Y (a) and PC12 (b) cells. The cells were treated with desired concentration of acetylshikonin for 12 hours and then stimulated with H_2_O_2_ (500 *μ*M) for 1 hour, and the apoptosis-related protein expression was detected by western blot.

To further explore the detailed neuroprotective mechanisms of acetylshikonin on H_2_O_2_-induced apoptosis, the possible related proteins were measured by western blot. As shown in Figures 4(a) and 4(b), H_2_O_2_-stimulation decreased Bcl-2 expression level, while it increased Bax and p53 expression level. In contrast, acetylshikonin concentration dependently led to increased expression of Bcl-2 as well as decreased Bax and p53 expression in H_2_O_2_-induced SH-SY5Y and PC12 cells. Caspase cascade has been identified as the critical executor for apoptosis. In H_2_O_2_-induced cells, the decreased caspase-3 and the increased cleaved caspase-3 were observed, which was rescued by acetylshikonin in a dose-dependent manner.

### 3.6. Upregulation of Heme Oxygenase-1 (HO-1) by Acetylshikonin Played a Key Role of Its Antiapoptotic Activity in H_2_O_2_-Induced SH-SY5Y Cells

**Figure 5:**
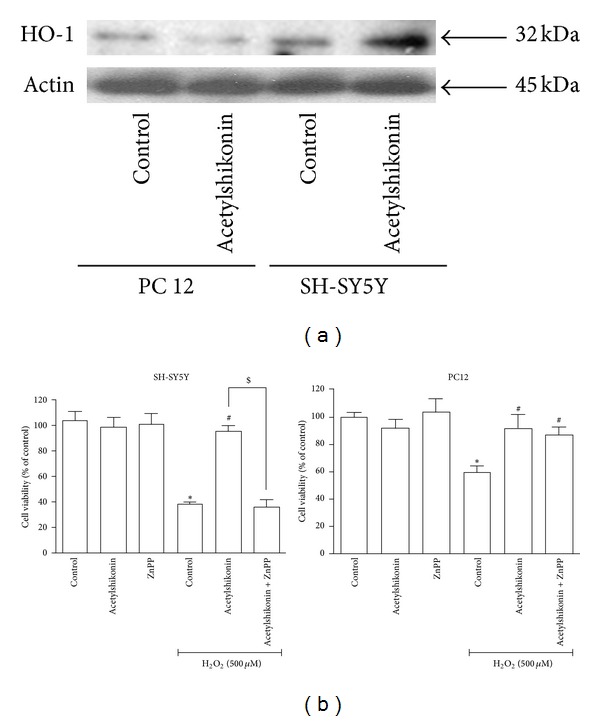
Antiapoptotic activity of acetylshikonin in H_2_O_2_ stimulation was related with induction of HO-1 expression in SH-SY5Y cells. (a) Both SH-SY5Y and PC12 cells were treated with desired concentration of acetylshikonin for 12 hours and then stimulated with H_2_O_2_ (500 *μ*M) for 1 hours, and HO-1 protein expression was detected by western blot. (b) SH-SY5Y or PC12 cells were pretreated with HO-1 inhibitor (ZnPP, 10 *μ*M) for 2 hours, then incubated with desired concentration of acetylshikonin for 12 hours, further stimulated with H_2_O_2_ (500 *μ*M) for 4 hours, and the cell viability (right panel: SH-SY5Y; left panel: PC12) was measured by MTT. Data shown are means ± SEM of results from independent experiments in triplicate. *P < 0.05 compared with control cells; ^#^ *P* < 0.05 compared with H_2_O_2_-stimulated cells; ^$^ *P* < 0.05 compared with acetylshikonin-treated cells.

It has been widely accepted that upregulation of Heme oxygenase 1 (HO-1) expression protects cells against the oxidative-stress cellular injury [23]. Western blot results showed that HO-1 expression was increased after acetylshikonin treatment in SH-SY5Y cells. However, acetylshikonin treatment has no impact on the level of HO-1 expression in PC12 cells (Figure 5(a)). To further confirm the role of HO-1 in antiapoptotic effects of acetylshikonin, we cotreated acetylshikonin with ZnPP (HO-1 inhibitors, 10 *μ*M) in H_2_O_2_-induced cells. Cell viability results demonstrated that ZnPP reversed the protective effects of acetylshikonin on H_2_O_2_-induced cell death in SH-SY5Y cells (Figure 5(b), right panel). However, these reversed effects of ZnPP were not observed in PC12 cells (Figure 5(b), left panel), and they were consistent with Western blot results. Therefore, HO-1 induction by acetylshikonin was critical against the oxidative-stress induced cell apoptosis in SH-SY5Y cells. In contrast, acetylshikonin was not able to upregulate HO-1 expression in PC12 cells, indicating that antiapoptotic activity of acetylshikonin in oxidative stress condition might be mediated through other antioxidant pathway in PC12 cells.

## 4. Discussion

With the rapid advances in personal computing power, virtual drug screening is popular and prominent. While there are numerous databases for synthetic compounds, there are only a few natural product databases that are specifically for *in silico* docking study. To facilitate virtual docking on natural compounds, we have established our in-house natural products database, which contains approximately 8,000 naturally occurring chemicals so far. Based on docking screening, top chemicals in ranking list were selected for the following analysis. The classic analysis, which has been widely accepted in most synthesis chemical virtual screen, is to rank the binding affinity and then choose the high ranking chemicals for further *in vitro *validation. Natural products, unlike synthesis chemicals, come from natural sources, such as plants, fungus, animals, and minerals. This characteristic might lead to a possibility that the high ranking chemicals have derivatives in the same species or genus. The collection of this kind of derivatives has been identified as bioactive fraction in complementary medicine. Therefore, we selected these top ranking derivatives for further validation. Here we identified three bioactive fractions as AChEIs, including anthraquinone fraction in RHEI RADIX ET RHIZOMA, furanocoumarin fraction in ANGELICAE DAHURICAE RADIX, and naphthoquinone fraction in ARNEBIAE RADIX. In this way, our database is not only suitable to screen pure compounds but also helpful to identify bioactive fractions. 

Ellman assay results showed that anthraquinones from RHEI RADIX ET RHIZOMA were the strongest AChEIs among these potential chemicals. In contrast, naphthoquinone from ARNEBIAE RADIX exhibited most potent attenuation activity against H_2_O_2_-induced apoptosis in both SH-SY5Y and PC12 cells. Particularly, acetylshikonin, one naphthoquinone from ARNEBIAE RADIX, not only significantly inhibited AChE activity but also dramatically rescued oxidative stress-induced apoptosis in both SH-SY5Y and PC12 cells. 

In addition to the role on the inhibition of Ach hydrolysis, there is evidence that all the FDA-approved AChEIs (tacrine, donepezil, rivastigmine, and galantamine) are neuroprotective agents. Three AChE inhibitors (tacrine, galanthamine, and donepezil) increased the activities of catalase (CAT) and glutathion peroxidase (GSH-Px) and protected PC12 cells from apoptosis generated by hydrogen peroxide. Donepezil also protected rat septal neurons from the toxicity induced by amyloid, while tacrine significantly attenuated hydrogen peroxide-induced injury and reversed hydrogen peroxide-induced overexpression of bax and p53 in PC12 cells [24–26].

Acetylshikonin, the major active components of ARNEBIAE RADIX, exhibit many biological effects including anticancer [27], antioxidant [28], and antiobesity [29]. Recent study also revealed that acetylshikonin might increase antioxidant enzyme activity and nitric oxide levels in ethanol-induced ulcer rat models [28]. Shikonin, the analogs of acetylshikonin, has been reported to protect PC12 against 6-hydroxydopamine-mediated neurotoxicity [30]. However, reports on the antioxidative stress effects of acetylshikonin on neuronal cells are limited.

Mitochondria have been identified as a key site of cell apoptosis and death. The dysfunction of mitochondria resulted in ROS generation as well as mitochondria membrane potential loss. The cleaved caspase-3 and PARP were upregulated by excessive ROS and mitochondria membrane potential loss, which subsequently triggered cell apoptosis [31, 32]. Present study demonstrated that acetylshikonin rescued H_2_O_2_-mediated ROS production, ΔΨm loss, and upregulation of cleaved caspase-3. Furthermore, H_2_O_2_ stimulation resulted in upregulation of Bax expression and downregulation of Bcl-2 expression, therefore leading to the decline of Bcl-2/Bax ratio that served as another important indicator of mitochondrial dysfunction [33]. Western blot results confirmed that acetylshikonin increased the Bcl-2/Bax ratio by upregulation of Bcl-2 and downregulation of Bax, indicating that H_2_O_2_-induced mitochondrial dysfunction might be attenuated by acetylshikonin. In addition, p53, another proapoptotic factor [34], is essential for H_2_O_2_-induced apoptosis in glioma cells. A high level of p53 expression was observed in H_2_O_2_-induced apoptosis; however, this apoptosis was significantly reduced by antisense p53 oligonucleotide [35]. The increased p53 has been inhibited by acetylshikonin. Together, acetylshikonin has been reported to protect mitochondrial function from oxidative stress in both SH-SY5Y and PC12 cells.

Upregulation of HO-1 is the major approach to prevent H_2_O_2_-induced cells from apoptosis and cell death [36]. For further mechanistic exploration, the expression level of HO-1 was detected by western blot. Results showed the upregulation of HO-1 by acetylshikonin was observed in SH-SY5Y cells but not in PC12 cells. In cell proliferation assay, the specific antagonist of HO-1 ZnPP abolished the protective effects of acetylshikonin in SH-SY5Y cells. Consistent with western blot results, ZnPP cannot exert its effect on the neuroprotective activity of acetylshikonin in PC12 cells. Notably, shikonin, the analogs of acetylshikonin, has been reported to induce the Nrf2-ARE system that might upregulate the transcription of HO-1 genes [37]. Further systematic studies are needed to investigate whether upregulation of HO-1 effect by acetylshikonin is mediated by Nrf2-ARE pathway.

## 5. Conclusion

Together, we first reported that acetylshikonin, a novel AChEI, exhibited antiapoptotic activity through an HO-1 dependent mechanism in SH-SY5Y cells. Therefore, the findings suggested that acetylshikonin might provide potential benefits for Alzheimer's diseases treatment.

## Figures and Tables

**Table 1:** Summary of ranking list of molecular docking screen.

Chemical name	Binding affinity	Binding residues (H-bond)	Binding residues (*π*-*π*)
Huperzine A	−10.4	Ser125, 203	Trp86, Tyr337
Galantamine	−8.4	Trp86, Tyr337, 124	Trp86, Tyr337
Tacrine	−8.4	Tyr337	Trp86
Emodin	−8	Tyr133, 337, Glu202	Trp86
Aloe-emodin	−8.2	Trp86, Ser125	Tyr337
Chrysophanol	−8		Trp86
Rhein	−7.4	Asp74, Tyr337	Trp86
Xanthotoxin	−8.5	Ser125	Trp86, Tyr337
Phellopterin	−8.5	Tyr337, 124, Ser125	Trp86
Alloisoimperatorin	−9.4	Tyr133, Asn87, Ser125	Trp86
Imperatorin	−8.2	Tyr337, 341, 124, Asp74	Trp86
Shikonin	−9.2	Glu202, Tyr337	Trp86
Acetylshikonin	−8.6	Ser203, Gly121, 122, 126	Trp86, Tyr124
Isovalerylshikonin	−8.1	Gly120, 126, Tyr337, Ser203	Trp86
*β*,*β*-Dimethylacrylshikonin	−8.5	Tyr337, Ser203, Gly120, 121, 122	Trp86

**Table 2:** Summary of potential AChE inhibitors from molecular docking screen.